# Uncover Hidden Physical Information of Soft Matter by Observing Large Deformation

**DOI:** 10.1002/advs.202414526

**Published:** 2025-04-07

**Authors:** Huanyu Yang, Yitao Cheng, Penghui Zhao, Jiageng Cai, Zhaowei Yin, Shaomin Chen, Ge Guo, Chi Zhu, Ke Liu, Lingyun Zu

**Affiliations:** ^1^ Department of Cardiology and Institute of Vascular Medicine Peking University Third Hospital Beijing 100191 China; ^2^ Department of Advanced Manufacturing and Robotics Peking University Beijing 100871 China; ^3^ State Key Laboratory of Vascular Homeostasis and Remodeling Peking University Beijing 100191 China; ^4^ NHC Key Laboratory of Cardiovascular Molecular Biology and Regulatory Peptides Peking University Beijing 100191 China; ^5^ Beijing Key Laboratory of Cardiovascular Receptors Research Beijing 100191 China; ^6^ Department of Radiology Peking University Third Hospital Beijing 100191 China; ^7^ Department of Mechanics and Engineering Science Peking University Beijing 100871 China; ^8^ State Key Laboratory of Transvascular Implantation Devices The Second Affiliated Hospital Zhejiang University School of Medicine 88 Jiefang Rd Hangzhou 310009 China; ^9^ Beijing Key Laboratory of Magnetic Resonance lmaging Devices and Technology Peking University Third Hospital Beijing 100191 China

**Keywords:** Bayesian optimization, finite element simulation, non‐destructive testing, soft matters

## Abstract

Accurate and non‐destructive detection of material abnormalities inside soft matter remains an elusive challenge due to its variable and heterogeneous nature, especially regarding non‐visual information. Here, a method is introduced that uncovers the physical information of internal material abnormalities from large deformations observed on the surface of the soft object. It finds the most probable values of imperceptible physical parameters by matching the nonlinear surface deformation between observation and finite element simulation through parallel Bayesian optimization, balancing the trade‐off between simulation accuracy and computational efficiency. Numerical and experimental tests, including simulated cases of aortic valve calcification, are conducted to showcase the effectiveness of our method, where we successfully recover hidden physical parameters including material stiffness, abnormality shape, and location. The method holds substantial promise for advancing the fields of material perception of robots, soft robotics, biology, and medical diagnostics, offering a powerful tool for the precise, efficient, and non‐invasive analysis of soft matter.

## Graphical abstract

Detecting internal abnormalities in soft matter remains challenging due to its heterogeneous nature. This study introduces a method that infers hidden physical properties by matching observed deformation with simulation through parallel Bayesian optimization. This method advances material perception in robotics, biology, and medical diagnostics.

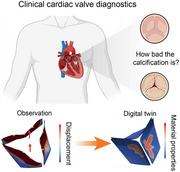

## 1 Introduction

Non‐destructive testing (NDT)^[^ ^1^, ^2^ ^]^ is a collection of techniques used to evaluate the integrity of material surfaces or internal flaws without causing damage. Typically, its goal is to identify abnormalities or material inconsistencies that could change the expected behavior of an object. Recently, many researchers have made efforts to estimate material properties to realize accurate NDT. Laser vibrometry^[^ ^3^ ^]^ and Digital Image Correlation (DIC)^[^ ^4^ ^]^ are effective tools for measuring surface displacements. Specifically, laser vibrometry can be used to inspect the integrity of structural buildings^[^ ^5^, ^6^ ^]^ and materials.^[^ ^7^, ^8^ ^]^ DIC^[^ ^9^, ^10^ ^]^ and vibrometry^[^ ^11^, ^12^ ^]^ have been applied as modal analysis techniques to infer the properties of homogeneous materials.^[^ ^13^, ^14^ ^]^ Due to the restricted operating conditions and complex setup of these sensors, these methods are often less accessible. As a result, visual testing methods present a practical and flexible alternative. In recent years, AI‐assisted approaches have been increasingly used for the characterization of materials' physical information. For instance, visual vibration tomography can be used to infer spatially‐varying Young's modulus and density,^[^ ^15^ ^]^ images can be utilized to estimate material types and surface properties,^[^ ^16^, ^17^, ^18^, ^19^, ^20^, ^21^ ^]^ and point clouds can facilitate material classification.^[^ ^22^, ^23^ ^]^ While the field is rapidly evolving due to high industrial demand, existing approaches remain insufficient for fully addressing the complexities of soft matter due to its variable and heterogeneous nature.

**Figure 1:**
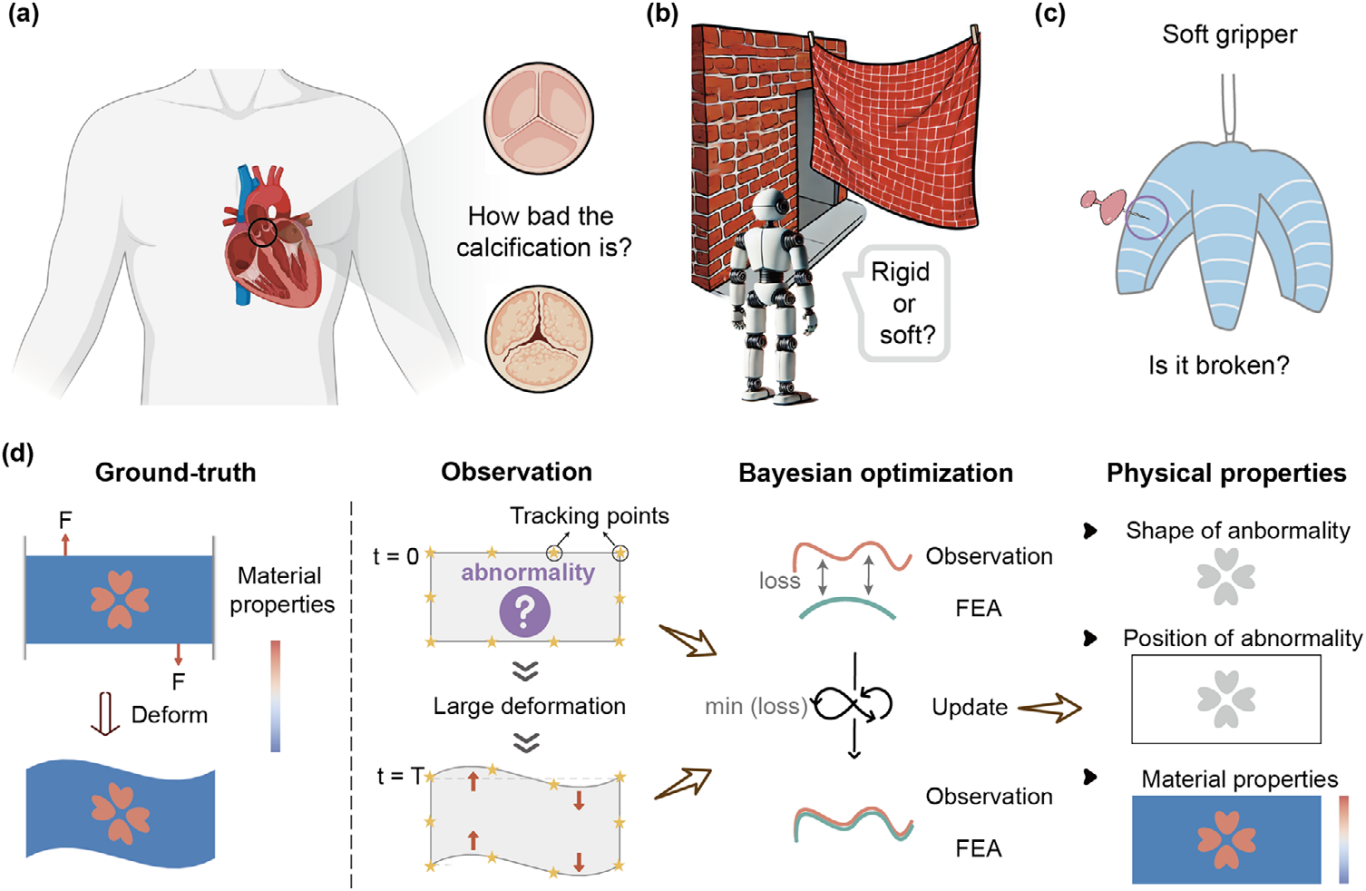
Method overview. In the fields of a) medical diagnostics, b) material perception of robots, and c) soft robotics, our method offers a powerful tool for non‐invasive analysis by observing surface deformation. d) For any object with material abnormalities, large deformations of key points, as observed on the object's surface over time serve as the input to the parallel Bayesian optimization algorithm, in which a virtual twin model based on finite element analysesis constructed. Through iterative updates, physical information such as the material properties, as well as the shape and location of the abnormalities can be accurately uncovered when the virtual and physical models match in observed deformation.

The non‐destructive or non‐invasive detection and analysis of soft matter are crucial for advancing fields such as medical diagnostics, material perception of robots, soft robotics^[^ ^24^ ^]^ and biology (**Figure** **1**a–c), particularly in assessing the internal structures and material properties. For instance, in the biomedical field, non‐invasive detection of in‐vivo soft tissues plays a vital role in fast and accurate diagnosis and treatment planning. A prominent example is calcific aortic valve disease (CAVD), the leading cause of aortic stenosis globally, which relies on qualitative visual inspection using echocardiography and/or multi‐slice computed tomography (MSCT).^[^ ^25^, ^26^, ^27^, ^28^ ^]^ However, conventional echocardiography often fails to accurately assess calcification due to noise,^[^ ^29^ ^]^ while the temporal resolution of MSCT is too low to capture the dynamic motion of valves.^[^ ^30^ ^]^ The limitations of these methods in providing a fully quantitative assessment of the pathology underscore the urgent need in clinical practice for more robust, physically‐informed, and detailed diagnostic approaches.

Typically, to understand the behavior of soft matter, numerical simulations are used as virtual twins of the physical objects.^[^ ^31^, ^32^, ^33^ ^]^ Numerous open‐source software tools have been developed specifically to model the complex multi‐physics processes in soft matter systems. For example, svFSI is a multiphysics finite element solver that enables the coupled simulation of electro‐mechanical and hemodynamic interactions, effectively modeling fluid dynamics, structural mechanics, electrophysiology, and their multiphysics interactions.^[^ ^34^, ^35^ ^]^ Other tools, like FEBio, openCARP, and those developed by Quarteroni et al. focus on structural mechanics, electrophysiology, and biomechanical modeling, providing precise outputs such as stress, strain, and displacement based on defined inputs.^[^ ^36^, ^37^, ^38^, ^39^ ^]^

Based on advanced finite element‐related studies, various techniques, such as the inverse finite element method (iFEM), have been developed to recover the physical properties of objects. Van Tonder et al.^[^ ^40^ ^]^ proposed an improved iFEM method for Mooney–Rivlin type material characterization, effectively addressing the challenge of stucking in local minima during optimization. Their constrained optimization technique significantly reduces parameter variation, thereby enhancing the accuracy and robustness of material property estimation. Additionally, many physics‐informed deep learning based methods have been developed. Pal and Naskar^[^ ^41^ ^]^ proposed a machine learning approach to predict stress–strain plots for Marlow hyperelastic material design, demonstrating the potential of data‐driven models in material characterization. Paral et al.^[^ ^42^ ^]^ introduced an ANN‐based damage identification method that leverages changes in the first mode shape profile to detect damage location and severity. Similarly, Zhong et al.^[^ ^43^ ^]^ developed a CNN‐based approach using mode shapes and mode curvature differences as inputs, where mode shapes provided damage localization, though mode curvature differences exhibited lower accuracy for detecting low‐degree damage. Perera et al.^[^ ^44^ ^]^ proposed a roaming damage method that employs artificial neural networks to predict unmeasured mode shape data using limited observations from bridge structures under various damage scenarios. Physics‐Informed Neural Networks^[^ ^45^ ^]^ have emerged as a promising deep learning framework for solid mechanics inversion and surrogate modeling by incorporating governing equations into the learning process. This hybrid approach enhances physical consistency, enabling parameter identification and bridging the gap between traditional numerical methods and purely data‐driven techniques. However, current approaches mainly rely on gradient‐based methods, which requires tedious sensitivity analysis, making them difficult to address various inverse problems, particularly in complex scenarios involving nonlinearity and large deformation.

By matching visible observations between the virtual and physical worlds, we create a method to recover soft matter's hidden physical information such as material properties, as well as the shape and location of internal abnormalities, based on the parallel Bayesian optimization algorithm and finite element modelling (Figure 1d). The deformation of an object is determined by its material distributions; conversely, the observed deformation of the object can be used to recover its inherent material properties. This interrelationship forms the basis of our physically informed NDT method. The large deformations observed on the soft matter's surface serve as the input, which can be obtained by standard echocardiography and/or MSCT. A finite element model is then built as the virtual twin to the physical object. By minimizing the difference between the surface deformation on the finite element model and the observed physical model, the physical parameters in the virtual model gradually approach the ground truth. However, this framework faces several challenges: difficulty in obtaining gradient during the minimization, extensive computation time per simulation, and large search spaces for physical parameters such as material moduli. Traditional optimization algorithms that require extensive evaluation of the forward model and its gradient are thus not suitable for our problem. We turn to the gradient‐free parallel Bayesian optimization algorithm,^[^ ^46^ ^]^ which is typically used to optimize functions that are costly to evaluate and are well‐suited for non‐convex inverse problems.

As a result of this approach, our method can accurately recover physical properties such as Young's moduli and identify the shape and position of material abnormalities that could alter the expected behavior of the object. We demonstrate our method on three types of numerical examples: the beam, inflation, and aortic valve model, each featuring distinct geometric features, boundary conditions, and abnormalities. To further validate our approach, we conduct experiments on a beam model, which demonstrates that our method has significant potential for precise, efficient, and non‐invasive inspections of soft matter. In addition, compared to most existing inverse finite element methods and physics‐informed deep learning‐based methods, our algorithm is specifically designed to handle nonlinear scenarios involving large deformations. By employing a gradient‐free Bayesian optimization algorithm, our method is capable of solving a wide range of problems that can be simulated using various approaches including finite element method, offering superior generalizability. Furthermore, our method is model‐free, unlike physics‐informed deep neural networks, which require extensive training data. We also leverage parallel computing to improve computational efficiency, which makes our approach scalable to larger‐scale problems and allows for accurate estimates within relatively short amount of time.

Our method holds promising applications in material perception of robots, soft robotics, biology, and medical diagnostics. Additionally, this method can be further utilized to construct virtual twin models of soft matter in the physical space, enabling real‐time monitoring, operational simulations, and state predictions. This underscores its considerable promise for future applications in robotic interaction and control, medical diagnostics, and treatment planning.

## 2 Results

### 2.1 Numerical Examples

Three numerical examples are conducted to validate our approach, each characterized by distinct geometry and boundary conditions. For each example, two models are prepared: one with abnormality and one without, in order to demonstrate the effect of internal abnormality on material deformation. We conduct four types of tests for each example with different unknown properties to validate the robustness and versatility of our proposed approach in handling diverse conditions and configurations. All simulations are conducted using svFSI.^[^ ^34^ ^]^

#### 2.1.1 Beam Bending Test

**Figure 2:**
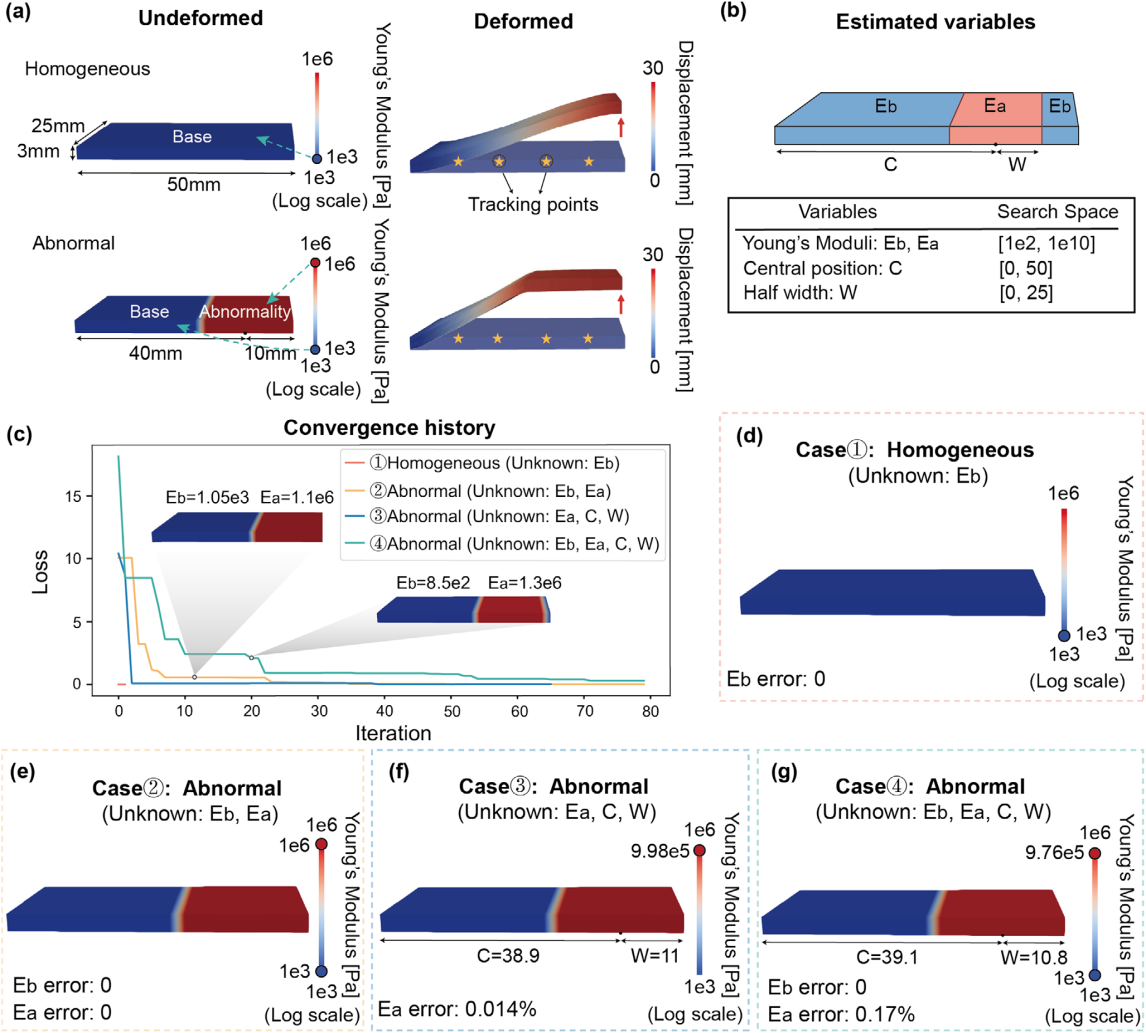
Results of the beam bending test. a) The undeformed and deformed configurations of the homogeneous and abnormal models. The blue and red dots on the color bar indicate the ground truth moduli of the base (*E*_*b*_) and abnormal (*E*_*a*_) parts. The tracking points are marked by yellow stars. The red arrow indicates the applied load. b) List of physical parameters to be estimated. c) Convergence history of the four test cases. The estimation results are shown in (d), (e), (f), and (g). d) Case 1: homogeneous material property without abnormality. e) Case 2: geometries are known, and material properties are to be estimated. f) Case 3: *E*_*b*_ is known, geometry and material properties of the abnormal part are to be estimated. g) Case 4: All physical parameters need to be estimated.

We start with a basic 3D beam example with dimensions of 50 mm × 25 mm × 3 mm, which is discretized into a uniform hexahedral mesh of 20 × 6 × 4 elements, for balance between accuracy and computational efficiency. The homogeneous model is made of a single type of material designed to mimic silicone rubber, with an initial tangent of Young's modulus *E*_*b*_ = 1 × 10^3^ Pa. For details of the nonlinear constitutive model, please refer to the Method section. The abnormal model is embedded with a brick‐shaped region of abnormality. The base material properties are identical to the homogeneous model, which is *E*_*b*_ = 1 × 10^3^ Pa, and the abnormal region differs by exhibiting a significantly higher Young's modulus *E*_*a*_ = 1 × 10^6^ Pa. The abnormal region measures 20 mm in length, spanning the beam's width and height, which is located on the right end of the beam as shown in **Figure** **2**a. We consider a homogeneous density of ρ = 1 kg/mm^3^ and Poisson's ratio of ν = 0.5 for both materials. The left end face of the beam is fixed, and a uniform upward displacement is applied to the right end face, which causes the right side to move upwards by 30 mm. Displacements of the tracking points from these two models with all physical parameters given are considered as the ground truth. An explicit finite element method was employed to compute the displacement field under the same boundary conditions.

Four evenly distributed points along the long edge of the beam are selected as the key points for tracking (Figure 2a). For the homogeneous model, our goal is to determine the value of the estimated Young's modulus ${\tilde{E}}_{b}$ of the base material. In the case of the abnormal model, we aim to not only estimate Young's moduli of both the base material ${\tilde{E}}_{b}$ and the abnormal material ${\tilde{E}}_{a}$ but also approximate the position and shape of the abnormality defined by center position *C* and half‐width *W*, as shown in Figure 2b. The search space for Young's moduli ${\tilde{E}}_{b}$ and ${\tilde{E}}_{a}$ spans from 1 × 10^2^ to 1 × 10^10^ Pa, accommodating the typical range for most materials. The parameters for the central position $\tilde{C}$ and half‐width $\tilde{W}$, are constrained within the ranges of [0, 50] mm and [0, 25] mm, respectively.

**Figure 3:**
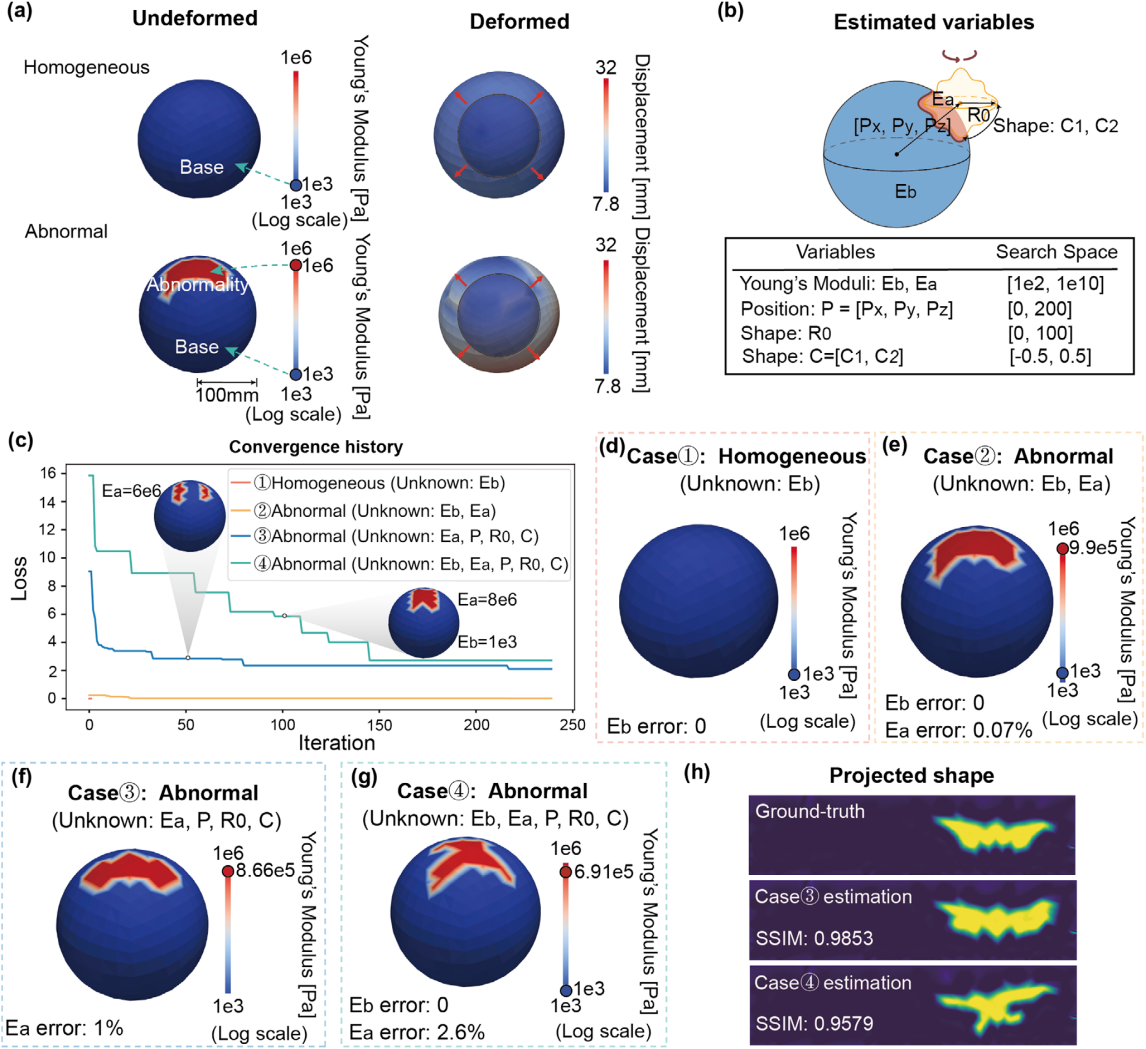
Result of the balloon inflation test. a) The undeformed and deformed configurations of the homogeneous and abnormal models. The blue and red dots on the color bar indicate the ground truth values of *E*_*b*_ and *E*_*a*_. The red arrow indicates the applied load. The tracking points are taken as all vertices of the finite element discretization. b) List of physical parameters to be estimated in this example. c) Convergence history of the four test cases. d–g) The estimation results of Cases 1 to 4. h) Spherical map projections of the northern hemisphere of the balloon, comparing the ground truth and estimated shapes of the abnormalities.

We select different types of unknowns and prepare four cases for testing, as illustrated in Figure 2c–g. Figure 2c shows the convergence history of minimizing the loss. The loss is defined as the deformation difference between the predefined benchmark model (or ground truth) and the model with unknowns, calculated based on the tracking points (see the Experimental Section for details). We observe a fast convergence in less than 20 iterations for all cases. For the homogeneous model, the precise value of ${\tilde{E}}_{b}=1\times 10^{3}\mathrm{Pa}$ is obtained just after one iteration (Figure 2d). For the abnormal model, we conduct three different tests: (1) estimate both ${\tilde{E}}_{b}$ and ${\tilde{E}}_{a}$ (Figure 2e); (2) estimate ${\tilde{E}}_{a}$ as well as its position *C* and shape *W* (Figure 2f); (3) estimate all physical parameters including ${\tilde{E}}_{b}$, ${\tilde{E}}_{a}$, *C*, and *W* simultaneously (Figure 2g). The three tests converge to the most likely values of material properties within no more than 80 iterations, with errors less than 0.2%. Moreover, our approach successfully recovered the position and shape of the abnormal region (see Movie S1, Supporting Information). During these tests, each iteration took approximately 10 min to simulate and update.

To further demonstrate the generality of our method, we introduce a new case, which shares the same geometry, boundary condition, and tracking points as the predefined benchmark model. However, the constitutive model for this case is set as the Saint Venant–Kirchhoff hyperelastic model^31^, ^61^. The displacement field calculated above is taken as the target for estimation. The estimated variables are the same as in case 3: the Young's modulus of the abnormal material ${\tilde{E}}_{a}$, along with the central position $\tilde{C}$ and half‐width $\tilde{W}$ of the abnormality. After 6 iterations, we get the nearly correct results: ${\tilde{E}}_{a}=1.04\times 10^{6}$, $\tilde{C}=4.07$, and $\tilde{W}=0.92$. The detailed optimization process and constitutive model are provided in the Supporting Information. This shows that our method can recover identical results even when applied to different models, highlighting its generalizability. Therefore, our method can be readily extended to problems involving more complex base models.

#### 2.1.2 Balloon Inflation Test

We develop a 3D isotropic balloon example with a radius of 100 mm and a thickness of 3 mm discretized into hexahedral elements to simulate an inflation process by applying pressure on the inner face. The ground truth of the material properties are set as: *E*_*b*_ = 1 × 10^3^ Pa, *E*_*a*_ = 1 × 10^6^ Pa (**Figure** **3**a), consistent with the previous example. The region of the abnormality is defined as the intersection of the balloon and a shape generator defined as the revolved shape of the following curve^[^ ^47^ ^]^:

(1) $$\begin{array}{cc} & R_{\theta }=R_{0}\left[1+C_{1}\cos\left(4\theta \right)+C_{2}\cos\left(8\theta \right)\right]\\ & x_{1}=R_{\theta }\cos\left(\theta \right)\\ & x_{2}=R_{\theta }\sin\left(\theta \right). \end{array}$$

This curve revolved around the *z*‐axis to create complex three‐dimensional axis‐symmetric geometries. In addition to the tunable parameters *R*_0_, *C*_1_, and *C*_2_, the center of this geometry can also be translated in the three dimensional space by *P* = [*P*_*x*_, *P*_*y*_, *P*_*z*_] from the origin. The region on the balloon that is inside the shape generator is defined as the abnormal region. Therefore, the shape and position of the abnormal region are completely defined by three shape parameters: *R*_0_, *C*_1_, *C*_2_, and three position parameters: *P*_*x*_, *P*_*y*_, *P*_*z*_. By randomly generating a set of these parameters, we create an irregularly shaped area of abnormality, as depicted in Figure 3b.

For the homogeneous model, the balloon is made of a single type of material, whose modulus is set as *E*_*b*_ = 1 × 10^3^ Pa. For the abnormal model, we set *E*_*b*_ = 1 × 10^3^ Pa, *E*_*a*_ = 1 × 10^6^ Pa, *R*_0_ = 41.2 mm, [*C*_1_, *C*_2_] = [0.47, −0.35], [*P*_*x*_, *P*_*y*_, *P*_*z*_] = [− 4.5, 62.1, 65.7] mm. Our goal is to estimate different combinations of these values when they are unknown, as shown in Figure 3d–g. The search space of estimated values is defined as: ${\tilde{E}}_{b}\in \left[1\times 10^{2},1\times 10^{10}\right]\mathrm{Pa}$, ${\tilde{E}}_{a}\in \left[1\times 10^{2},1\times 10^{10}\right]\mathrm{Pa}$, ${\tilde{R}}_{0}\in \left[0,100\right]$ mm, ${\tilde{C}}_{1},{\tilde{C}}_{2}\in \left[-0.5,0.5\right]$, and ${\tilde{P}}_{x},{\tilde{P}}_{y},{\tilde{P}}_{z}\in \left[0,200\right]$ mm.

Figure 3c shows the convergence history of the four tests. For the homogeneous model, we can find the true value of ${\tilde{E}}_{b}=1\times 10^{3}$ Pa in one iteration (Figure 3d). As for the three tests of the abnormal model, the position and shape of the abnormality are recovered after no more than 250 iterations (Figure 3e–g), with quite high structural similarity (SSIM).^[^ ^48^ ^]^ Moreover, we find that Young's modulus in the base part ${\tilde{E}}_{b}$ can be accurately estimated no matter what the unknowns are. The slight mismatch of ${\tilde{E}}_{a}$, no more than 2.6% difference from the true *E*_*a*_, is attributed to the minor differences in the estimated shape of the abnormality. We show the spherical map projections of the northern hemisphere of the balloon in Figure 3h. To assess the accuracy of the shape estimation, we adopt a quantitative metric, the SSIM between the ground truth and the estimated shapes of the abnormality. When two shapes are identical, the SSIM becomes 1. The SSIM we obtained is 0.9853 and 0.9579 for cases 3 and 4, respectively. We note that such deviation is almost inevitable due to the nature of the problem that different combinations of the physical parameters may lead to the same deformation. Each iteration during the balloon inflation test required about 15 min for simulation and updates. The convergence process is shown in Movie S1 (Supporting Information).

#### 2.1.3 Aortic Valve Test

To demonstrate the potential application of our method, we design an example of the tricuspid aortic valve (TAV), simulating the motion of both a healthy valve and a CAVD‐afflicted valve over a single cardiac cycle. The healthy valve and the calcified valve are analog to the homogeneous model and the abnormal model in the previous examples, where the calcified part is considered as the abnormality. Using the general parametric geometry of the TAV,^[^ ^49^ ^]^ which is based on CT measurements, we construct a full 3D geometry of the aortic valve without calcification. The valve geometry is segmented into triangular shell elements.

**Figure 4:**
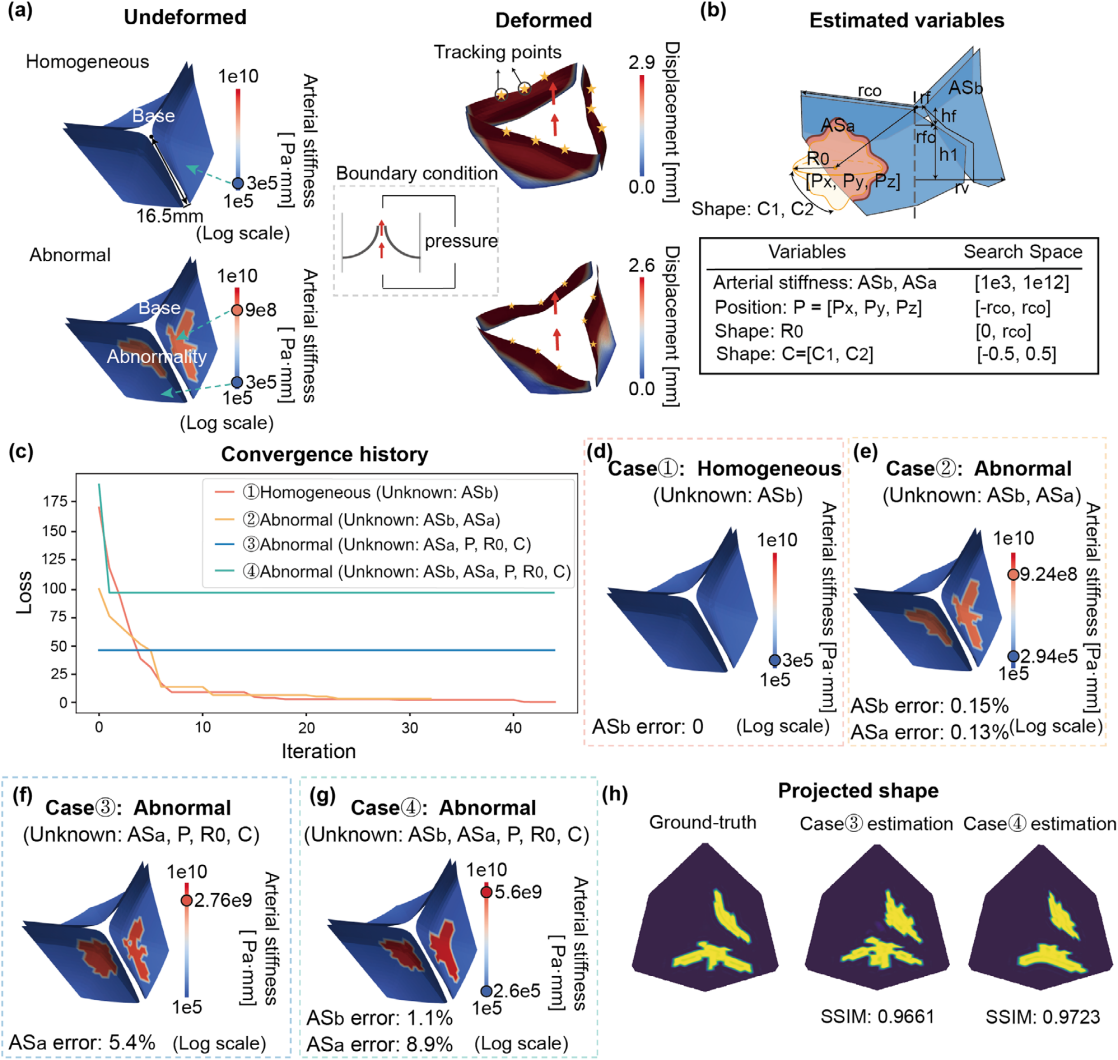
Results of the aortic valve test. a) The undeformed and deformed configurations of the homogeneous (healthy) and abnormal (calcified) valve models. The blue and red dots on the color bar indicate the ground truth values of *AS*_*b*_ and *AS*_*a*_. The tracking points are marked by yellow stars. The boundary condition is illustrated in the inset. b) Geometry of the aortic valve model, and a list of physical parameters to be estimated in this example. c) Convergence history of the four testing cases. d–g) The estimation results of Cases 1 to 4. h) Visualizations of the ground truth and the estimated shapes of the abnormal part using PCA to project the 3D models onto a 2D plane.

For the healthy valve model, Young's modulus *E*_*b*_ is set at 1 × 10^6^ Pa with a thickness *T*_*b*_ of 0.3 mm, reflecting typical physiological values. For the calcified parts in the CAVD model, Young's modulus *E*_*a*_ increases to 1 × 10^9^ Pa, with the thickness *T*_*a*_ expanding to 0.9 mm, aligning with average empirical values.^[^ ^50^ ^]^ To simplify, we represent the material properties of the valve using arterial stiffness, *AS* = *E* · *T*. The region of calcification is defined using the same shape generator as in the previous example, which could closely represent the partial arc shapes of typical calcification.^[^ ^50^ ^]^ Regarding boundary conditions, a trans‐valvular pressure gradient is applied across the valve. The lower edges of each leaflet are fixed, whereas the upper edges remain free, allowing the valve to mimic the in‐vivo response to cardiac pressures. For each valve, three points with the largest deformation in the upper half of the valve are selected as tracking points (**Figure** **4**a).

The calcified region is characterized by the shape and position parameters of the shape generator: *R*_0_, *C*_1_, *C*_2_, and [*P*_*x*_, *P*_*y*_, *P*_*z*_]. To estimate these unknown values, the ranges are defined as ${\tilde{E}}_{b}\in \left[1\times 10^{3},1\times 10^{12}\right]\;\mathrm{Pa}$, ${\tilde{E}}_{a}\in \left[1\times 10^{3},1\times 10^{12}\right]\;\mathrm{Pa}$, ${\tilde{T}}_{b}\in \left[1\times 0.3,1\times 10^{1.2}\right]\;\text{mm}$, ${\tilde{T}}_{a}\in \left[1\times 0.3,1\times 10^{1.2}\right]\;\text{mm}$, ${\tilde{R}}_{0}\in \left[0,r_{co}\right]$, ${\tilde{C}}_{1},{\tilde{C}}_{2}\in \left[-0.5,0.5\right]$, and ${\tilde{P}}_{x},{\tilde{P}}_{y},{\tilde{P}}_{z}\in \left[-r_{co},r_{co}\right]$, where *r*_*co*_ is the radius of the whole aortic valve.

Figure 4c illustrates the detection process for four types of tests. For the homogeneous model, the precise values of Young's modulus and thickness are accurately recovered after 40 iterations (Figure 4d). For the abnormal cases, when the shape and position of abnormalities are known, arterial stiffness for both the healthy and abnormal regions is estimated to closely match the ground truth (Figure 4e). Such a case is reminiscent of the situation when MSCT located the region of calcification. In case 3, when nothing is known except for the arterial stiffness of the healthy valve, the calcified arterial stiffness, correct positions, and highly similar shapes of the abnormalities are approximated after no more than 2 iterations (Figure 4f). In case 4, when no physical information is known, we also get pretty close estimates (Figure 4g) ‐ this mimics the situation when only ultrasonic videos are available. In all four tests, the differences in arterial stiffness for both the healthy and abnormal regions do not exceed 9%.

The small deviations in arterial stiffness arise due to subtle differences in the estimated shape of the abnormalities (Figure 4f,g). Specifically, we use the principal component analysis (PCA)^[^ ^51^ ^]^ algorithm to project the ground truth and the estimated results of cases 3 and 4 onto a two‐dimensional plane for visualization, as shown in Figure 4h. The SSIM between the 2D visualization results of cases 3 and 4 and the ground truth are 0.9661 and 0.9723, respectively. The accuracy of estimation can be further improved by considering more tracking points. In the aortic valve example, the simulation and update process for each iteration took approximately 8 hours, owing to the complexity of the problem. The convergence process is shown in Movie S1 (Supporting Information).

### 2.2 Experimental Example

To show the applicability of our method to practical problems, we design an experimental beam bending test (see Movie S2, Supporting Information). The elastic beam is prepared using Polydimethylsiloxane (PDMS).^[^ ^52^ ^]^ Preparation for PDMS is straightforward: the silicone base and curing agent are mixed by weight, the mixture is degassed to remove bubbles, poured over a master mold, and then cured in a vacuum oven to remove any entrapped gases. After cooling, the PDMS can be easily peeled and cut to shape.

**Figure 5:**
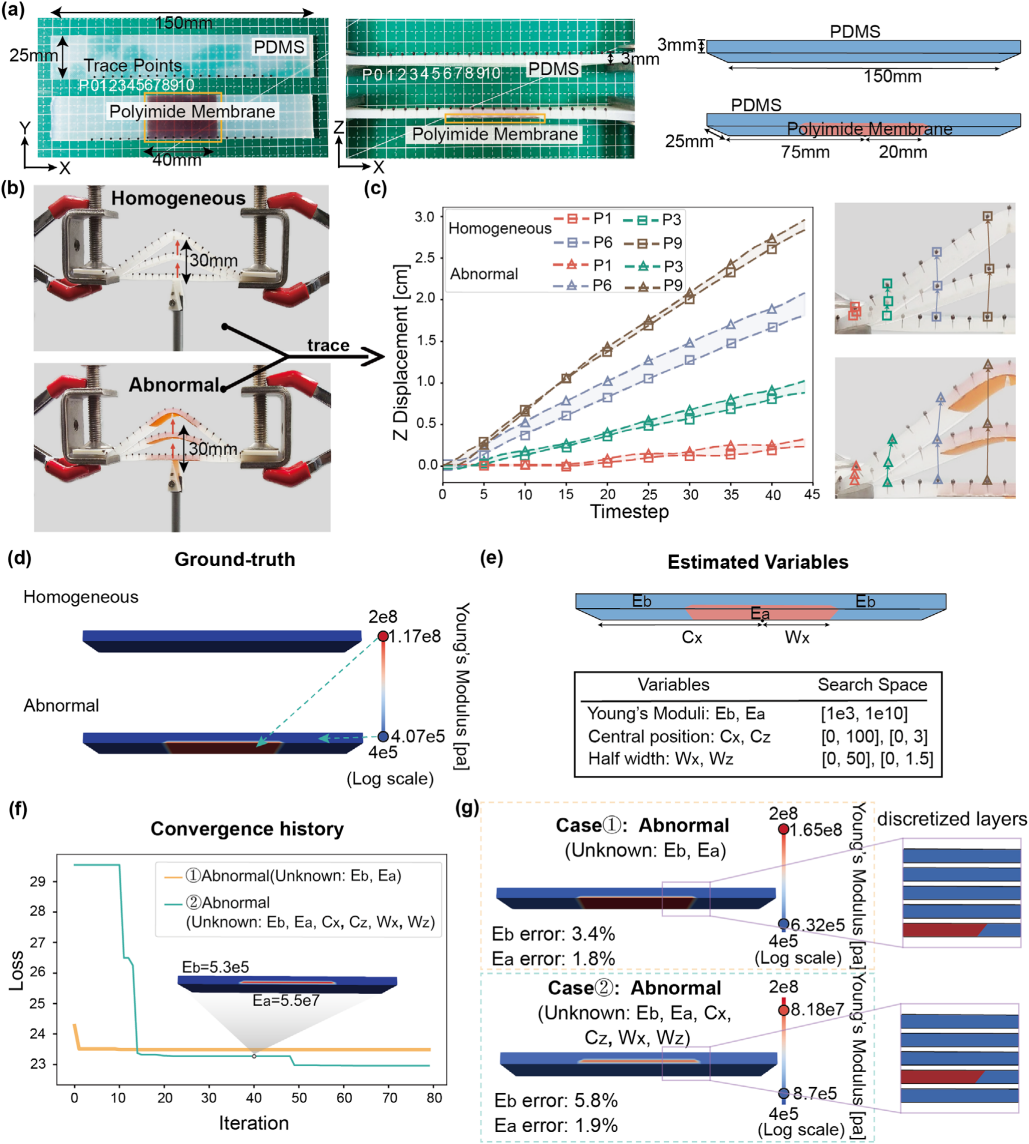
Results of the experimental test. a) A physical beam model is prepared using PDMS, with or without Polyimide membrane. Nineteen pins are nailed on the long edge of each model for visual tracking. b) The experimental setup. The left and right ends are securely fixed, and an upward displacement of 30mm is applied at the center of the beam. c) The *z*‐direction displacement of the tracking points over time for both models. d) The ground truth of both models. e) The geometry of the beam and the physical parameters are to be estimated. f) Convergence history of the two testing cases. g) The estimated results of the two cases. Case 1: geometries are known, and material properties are to be estimated. Case 2: all physical parameters of the abnormal model need to be estimated, including the vertical position of the Polymide membrane.

Two beam models, each measuring 100 mm × 25 mm × 3 mm, are fabricated using PDMS. One model is homogeneous, while the other includes a manually inserted 40 mm × 25 mm polyimide membrane at the bottom‐center of the mold during the PDMS casting process. Nineteen small pins are nailed evenly along the long edge to serve as visual tracking points, which are sufficiently contrastive and small to have little impact on tracking accuracy (**Figure** **5**a). The beam is designed to be symmetric about the *y*‐axis. An upward displacement of 30 mm is applied at the center along the *z*‐direction of the beam (Figure 5b). Due to its symmetry, we only need to simulate half of the beam for simplicity.

Using the binocular tracking algorithm mentioned in the Supporting Information, we take two videos simultaneously of the beam from different perspectives. The displacements of the tracking points are then calculated through the principle of binocular stereoscopic vision.^[^ ^53^, ^54^ ^]^ As shown in Figure 5b, the deformations of the homogeneous and the abnormal beams are quite different. Notably, the differences at tracking points 6 to 9, which are within the range of the abnormality, are particularly significant (Figure 5c). The displacements in the *xy*‐direction are too minor to be included in our consideration.

From previous numerical examples, we observe that when there is a single unknown modulus, the estimation made by our method is precise. Therefore, in this example, we estimate the effective initial Young's modulus of the base material as *E*_*b*_ = 4.07 × 10^5^ Pa using the homogeneous model, which is considered as the ground truth. For the abnormal part, given the ground truth *E*_*b*_ and the position of abnormality, we obtain the equivalent *E*_*a*_ = 1.17 × 10^8^ Pa (Figure 5d) as ground truth. Two cases of the test are then conducted: (1) estimation of ${\tilde{E}}_{b}$ and ${\tilde{E}}_{a}$; (2) estimation of ${\tilde{E}}_{b}$, ${\tilde{E}}_{a}$, and the shape and position of the abnormality, characterized by *C*_*x*_, *C*_*z*_, *W*_*x*_, and *W*_*z*_ (Figure 5e). In case 1, the estimated Young's modulus for the two regions is ${\tilde{E}}_{b}=6.32\times 10^{5}$ Pa and ${\tilde{E}}_{a}=1.65\times 10^{8}$ Pa. In case 2, we obtain ${\tilde{E}}_{b}=8.7\times 10^{5}$ Pa and ${\tilde{E}}_{a}=8.18\times 10^{7}$ Pa. The estimated geometry of the abnormality is shown in Figure 5g. In all tests, the error in the estimated Young's modulus does not exceed 6%.

In fact, the final estimation with all physical parameters unknown is more realistic. This is, because during the curing process, PDMS resin could flow underneath the polyimide membrane, which means that it cannot remain strictly at the bottom of the beam, but slightly above the bottom, as our estimation uncovered. The comparison of key point trajectories between the experimental and numerical results is illustrated in the Supporting Information.

In this example, there are three types of imperfections: noise in the observations, inaccuracies in the boundary and initial conditions, and errors in the model. Specifically, during the visual tracking process, inherent errors and noise are inevitable. Furthermore, due to sliding effects, the boundary conditions between the simulation and the experimental setup do not fully align. Model inaccuracies also exist, such as slight discrepancies between the actual beam length and the simulated length. However, despite these challenges, the overall results remain satisfactory. This is due to, unlike gradient‐based methods, which may sometimes diverge due to small errors, the Bayesian optimization algorithm ensures that predictions stay closer to reality by minimizing error at each iteration. By employing the Bayesian optimization algorithm, we are able to consistently bring the results closer to the true physical properties, even under imperfect conditions.

## 3 Conclusion

In this paper, we introduced a method for accurately uncovering the hidden physical information of soft matters by analyzing large deformations observed on the surface. Our approach utilizes a parallel Bayesian optimization algorithm combined with finite element simulations to minimize the difference in deformations observed in the virtual twin model and the actual physical model. Through three numerical examples and one experimental validation, we demonstrated the efficiency and accuracy of our approach in estimating material properties, abnormality shapes, and positions.

Our method differs significantly from existing studies in terms of target, solution algorithm, and effectiveness. Most existing research focuses on small deformations and is primarily targeted at infinitesimal deformations, whereas our method is designed to handle finite, nonlinear, and large deformations, making it more applicable to complex real‐world scenarios. Unlike traditional gradient‐based methods, we employ a gradient‐free Bayesian approach, which enhances generalizability and avoids the limitations of local minima. Moreover, our method achieves highly accurate estimations with significantly fewer iterations, leading to a rapid reduction in error. Especially in large deformation scenarios, the deformation discrepancy remains low. Additionally, our method takes advantage of parallel computing, which is not feasible for traditional gradient‐based approaches, further improving computational efficiency and scalability.

Our research has significant potential to advance the field of non‐destructive testing for soft matters, particularly in material perception of robots, soft robotics, biology, and medical diagnostics. For instance, the balloon inflation test is primarily applied in soft robot diagnostics. Many pneumatic soft robots rely on inflation to operate, and when these robots experience damage or material degradation, their behavior closely resembles the deformation patterns observed in the balloon inflation test. Our approach offers an effective method to assess the service condition of soft robots, detecting damage and degradation without the need for intrusive measures. The aortic valve test is primarily applied in clinical cardiac valve diagnostics. Our method can provide quantitative information to the doctors, as opposed to relying solely on qualitative judgment. We plan to apply our method for actual clinical tests in our future research.

Looking forward, integrating our algorithm with material perception of robots will provide necessary information about scene understanding, affordance estimation, manipulation, and grasp, realizing the potential for revolutionizing robotic interaction with the real world, and helping robots go beyond their current limits and reach their potential. Furthermore, using our method in the field of medical imaging techniques could lead to more quantitative, reliable, and accurate diagnostics for various internal diseases. The robust and adaptable performance of our approach promises great potential for applications in outcome prediction, prognosis, and digital twins in clinical settings. This paves the way for further innovations in medical diagnostics, offering a promising tool for medical practitioners and researchers.

## 4 Experimental Section

We aim to use the observed surface deformation to recover physical information of material abnormalities realizing NDT. The input for our method is a video capturing the surface deformation, for which a corresponding geometry is already known. The output includes values of physical properties, as well as the shape and position of abnormalities. This process involves three stages: 1) tracking the displacements of key points on the object's surface, 2) constructing the virtual twin model that is consistent with real scenarios, and 3) estimating physical information that best matches the observed deformation using our algorithm. Each iteration of the Bayesian optimization algorithm involves simulating finite element models in parallel to significantly enhance accuracy and efficiency. (Figure 1 shows an overview of the method).

### Estimating Material Properties

Our primary goal is to align the deformation calculated from finite element simulations with those tracked in experiments. The “mipego” python library is employed as our optimization Section Bayesian Optimization Algorithm. A finite element simulator is integrated and executed within the objective function at each iteration.

The experimental deformation of the key points is denoted as *D*. The corresponding numerical displacement is extracted as $\hat{D}$. Subsequently, the objective function is constructed by comparing the numerical deformation with the actual measurements and is formulated as:

(2) $$L={\left\|D-\hat{D}\right\|}^{2}$$

In practice, the algorithm simultaneously seeks the optimal physical information such as material properties *M* as well as the positions and shape of abnormalities, represented by *P* and *S*, respectively:

(3) $$M^{*},P^{*},S^{*}=arg\min_{M,P,S}L$$

### Bayesian Optimization Algorithm

Bayesian optimization (BO) is a strategy used for optimizing functions that are costly to evaluate, often requiring minutes or hours per assessment. It primarily involves two components: a Surrogate Model and an Acquisition Function. 1) Surrogate Model: BO begins by constructing a surrogate model of the objective function, which approximates the true function. This model helps quantify the uncertainty in predictions, typically employing Gaussian process regression. However, other Bayesian machine learning techniques or decision tree models may be employed based on the specific requirements and characteristics of the data. 2) Acquisition Function: This function, derived from the surrogate model, plays a key role in guiding the optimization process by determining where to sample next. It manages the trade‐off between exploitation (sampling where the model predicts high performance) and exploration (sampling in regions of high uncertainty). Common acquisition functions include Expected Improvement, Probability of Improvement, and Upper Confidence Bound. Each new sample updates the surrogate model, enhancing prediction accuracy and minimizing uncertainties, thus progressively refining the optimization approach.^[^ ^46^ ^]^

In this paper, we use the Mixed‐Integer Parallel Efficient Global Optimization(MIP‐EGO) algorithm^[^ ^55^ ^]^ to address the physical information recovering problem. The MIP‐EGO algorithm introduces significant improvements to the traditional BO approach:

(1) Moment‐Generating Function of the improvement: MIP‐EGO employs the so‐called Moment‐Generating Function (MGF)^[^ ^56^ ^]^ based acquisition function, which effectively balances exploitation and exploration. This function has a closed form and can be expressed as:
(4) $$\begin{array}{cc} & M\left(x,t\right)=\Phi \left(\frac{L_{min}-{\hat{L}}^{\prime }\left(x\right)}{s(x)}\right)e^{\left(L_{min}-\hat{L}\left(x\right)-1\right)t+\frac{{\hat{s}}^{2}\left(x\right)}{2}t^{2})}\\ & {\hat{L}}^{\prime }\left(x\right)=\hat{L}\left(x\right)-{\hat{s}}^{2}\left(x\right)t \end{array}$$
  where *L*_*min*_ = min {*y* ^(1)^, *y* ^(2)^, …, *y* ^(*n*)^} represents the current best performance over all evaluated configurations, $\hat{L}\left(x\right)$ denotes the prediction on configuration *x*, ${\hat{s}}^{2}\left(x\right)$ signifies the empirical variance of the prediction, and Φ(·) denotes the cumulative distribution function of the standard normal distribution. The acquisition function *M* incorporates an additional real parameter *t* (temperature), which is sampled from a log‐normal distribution *Lognormal*(0, 1) and directly influences the balance between exploring new regions and exploiting known areas of the search space. As the parameter *t* increases, *M* prioritizes configurations with high uncertainty, thereby enhancing exploration. Conversely, decreasing *t* shifts the focus toward exploitation, emphasizing configurations predicted to offer high performance. The value of *t* can be strategically set based on the available evaluation budget: a larger budget allows for a higher *t* setting, promoting a broader, albeit slower, global search strategy; a smaller budget suggests a lower *t* setting, which accelerates the search but focuses it more narrowly.
(2) Parallelization: MIP‐EGO facilitates parallel execution through batch‐sequential optimization, generating multiple candidate configurations in each iteration using different temperature parameters *t*. This leads to the instantiation of *q* different *M* functions based on the varied temperatures, each criterion aimed at identifying a promising configuration by maximizing its value. This not only accelerates the convergence process but also broadens the exploration of the solution space, making the algorithm more efficient in finding optimal configurations across a range of possible solutions.
  These enhancements allow MIP‐EGO to improve the optimization of complex systems significantly, offering faster convergence and broader exploration capabilities compared to traditional BO methods.[Boxed-text advs11891-fea-0001]

Algorithm 1 **MIP‐EGO for NDT.**

**Table:** 

1:	Construct finite element model
2:	Place Random Forest prior
3:	Sample the initial configuration at *n*_0_ points (*X*, *L*(*X*))
4:	Train a random forest (RF) on (*X*, *L*(*X*))
5:	**while** simulation ≠ observation or *n* ⩽ *N* **do**
6:	**for** each *i* = 1 → *q* **do**
7:	⊳ parallel for‐loop
8:	*t*_*i*_ ← *LognormalN*(0, 1)
9:	${\tilde{x}}_{i}$ ← *mies*(*x*, *t*_*i*_), *x* ∈ *C*
10:	compute ${\tilde{y}}_{i}\leftarrow L\left({\tilde{x}}_{i}\right)$
11:	**end for**
12:	$X\leftarrow X\cup \left\{{\tilde{x}}_{1},\text{…},{\tilde{x}}_{q}\right\}$
13:	$L\leftarrow L\cup \left\{{\tilde{y}}_{1},\text{…},{\tilde{y}}_{q}\right\}$
14:	re‐train RF on the augmented data set (*X*, *L*(*X*))
15:	**end while**

Considering the large search space of material properties, we choose random forest as our surrogate model out of its robust performance in handling complex and multi‐dimensional data. Our algorithms were executed on a high‐performance computing platform equipped with dual AMD EPYC 7H12 64‐core Processors, providing a total of 256 logical processors with a base clock speed of 2.60 GHz, and 503 GiB of DDR4 RAM. To accommodate computational demands, we set the number of candidate solutions and the number of allowable jobs for parallelizing to 100 each. The acquisition function employed is the MGF Infill with the temperature parameter *t* set to 2, facilitating an effective balance between exploration and exploitation.

### Constitutive Model

The mechanical behavior of soft materials is often described using hyper‐elastic material constitutive relations, particularly within the Ogden series, which includes models such as the Mooney–Rivlin and Neo–Hookean models.^[^ ^57^, ^58^, ^59^, ^60^ ^]^ In this study, we adopted the Neo–Hookean model for all three tests. The classical strain energy density function for this model is expressed as:

(5) $$\begin{array}{cc} & \phi =\frac{\mu }{2}\left(\lambda_{1}^{2}+\lambda_{2}^{2}+\lambda_{3}^{2}-3\right) \end{array}$$

where μ is the shear modulus, and the λ_*i*_ are the principal stretches.

For an incompressible Neo–Hookean material with *J* = λ_1_λ_2_λ_3_ = 1, the Cauchy stress tensor σ is expressed as:

(6) $$\begin{array}{cc} & \sigma =FSF^{T}-pI \end{array}$$

where **F** is the deformation gradient tensor, **I** is the identity tensor, and *p* represents the pressure, which is introduced to maintain the incompressibility condition *J* = 1. The second Piola–Kirchhoff stress tensor **S** is given by:

(7) $$\begin{array}{cc} & S=\mu (\lambda_{1}n_{1}⊗n_{1}+\lambda_{2}n_{2}⊗n_{2}+\lambda_{3}n_{3}⊗n_{3}) \end{array}$$

where **n**_*i*_ are the unit vectors along the principal directions of stretch.^[^ ^61^ ^]^

The displacement‐based governing equations for the materials are formulated as:

(8) $$\begin{array}{cc} & \rho \frac{d^{2}u}{dt^{2}}=\nabla \cdot \sigma \left(u\right)+\rho b\\ & J=1 \end{array}$$

where **u** represents the displacement, and **b** denotes the body forces.

In our approach, Young's modulus *E* is treated as an estimated variable and serves as the input parameter for the finite element model. We consider incompressible materials for all examples, and thus the Poisson's ratio is ν = 0.5. The relationship between the shear modulus μ and Young's modulus *E* is as follows:

(9) $$\begin{array}{c} E=2\mu (1+\nu ) \end{array}$$

## Conflict of Interest

The authors declare no competing interests.

## Author Contributions

H.Y., Z.L., K.L., and Z.C. designed the research. H.Y., Y.C., Z.P., and J.C. performed all experiments. P.Z., J.C., Z.Y., S.C., and G.G. performed data acquisition and analysis. H.Y. conducted the theoretical derivations and numerical simulations. K.L. supervised the project. All the authors participated in the analysis of the results and in the writing of the paper.

## Supporting information

Supporting Information

Supplemental Movie S1

Supplemental Movie S2

## Data Availability

The data that support the findings of this study are available from the corresponding author upon reasonable request.
