# Supplementary material for: Uncover Hidden Physical Information of Soft Matter by Observing Large Deformation
Source: Adv Sci (Weinh). 2025 Apr 7;12(21):2414526. doi: 10.1002/advs.202414526 (PMC12140332; doi:10.1002/advs.202414526)
Supplement: Supplementary file 1 — Supporting Information [file ADVS-12-2414526-s001.pdf]

ADVANCED SCIENCE
Open Access

Supporting Information

for *Adv. Sci.*, DOI 10.1002/advs.202414526

Uncover Hidden Physical Information of Soft Matter by Observing Large Deformation

Huanyu Yang, Yitao Cheng, Penghui Zhao, Jiageng Cai, Zhaowei Yin, Shaomin Chen, Ge Guo, Chi Zhu, Ke Liu* and Lingyun Zu**

Supporting Information:

Uncover Hidden Physical Information of Material Abnormalities in Soft Matters by Observing Large Deformation

Huanyu Yang[1,2], Yitao Cheng[2], Penghui Zhao[1,5,6,7], Jiageng Cai[1,5,6,7] Zhaowei Yin[1,5,6,7], Shaomin Chen[1,5,6,7], Ge Guo[8], Chi Zhu[4]*, Ke Liu[2,3]*, Lingyun Zu[1,5,6,7,9]*

*Corresponding author: Chi Zhu, Ke Liu, Lingyun Zu

[1] Department of Cardiology and Institute of Vascular Medicine, Peking University Third Hospital

[2] Department of Advanced Manufacturing and Robotics, Peking University

[3] State Key Laboratory of Transvascular Implantation Devices, The Second Affiliated Hospital Zhejiang University School of Medicine, 88 Jiefang Rd, Hangzhou 310009, P.R. China

[4] Department of Mechanics and Engineering Science, Peking University

[5] State Key Laboratory of Vascular Homeostasis and Remodeling, Peking University

[6] NHC Key Laboratory of Cardiovascular Molecular Biology and Regulatory Peptides, Peking University

[7] Beijing Key Laboratory of Cardiovascular Receptors Research. Beijing 100191, China

[8] Department of Radiology, Peking University Third Hospital, Beijing 100191

[9] Beijing Key Laboratory of Magnetic Resonance lmaging Devices and Technology, Peking University Third Hospital Beijing 100191, China

SUPPORTING NOTES

This PDF file includes:
Supporting Note 1: Motion Tracking
Supporting Note 2: Aortic Valve Model
Supporting Note 3: Spatial Abnormality Generator
Supporting Note 4: Estimation of ground-truth in experimental examples
Supporting Note 5: Additional beam bending test
Supporting Figs. S1 to S4
Other Supporting Information for this manuscript:
Movie 1: Numerical Examples
Movie 2: Experimental Example

Supporting Note 1: Motion Tracking

Visual tracking challenges are commonly addressed using monocular videos, which offer only two-dimensional information in the absence of explicit models for object shape or motion[1]. This limitation can be mitigated by employing binocular information through stereoscopic vision technology. Stereoscopic vision allows for the perception of depth on object points in 3D by capturing images from two distinct viewpoints with cameras positioned at a known baseline distance. Traditional 3D scene reconstruction involves processes such as epipolar rectification, feature detection, and triangulation, which involve matching corresponding points from the dual camera views. This method enhances the dimensional accuracy and depth perception crucial for comprehensive scene analysis and tracking[2].

We establish a binocular tracking system utilizing two cameras. Initially, a checkerboard is used as a standard calibration object, and at least 15 images are taken at various positions, angles, and orientations. This calibration process is crucial for determining the camera poses, which include the intrinsic matrix K, the rotation matrix R, the translation vector T, and the distortion coefficients[3]. After calibration, videos capturing the surface movement of the target object are recorded. For ease of tracking, contrast-enhanced spots are positioned at key points on the object. The camera poses to enable the projection of image pixel coordinates $u_1 = [u_l, v_l]^T$, $u_2 = [u_r, v_r]^T$, captured from two different perspectives, into 3D coordinates $X = [x, y, z]^T$ while simultaneously correcting camera lens distortion. With these definitions, the mathematical transformation of the traced i-th key point is given by the following equations:

$$\begin{aligned} z_i &= \frac{f \cdot T}{u_l - u_r} \\ x_i &= \frac{(u_l - c_x) \cdot z_i}{f}, \\ y_i &= \frac{(v_l - c_y) \cdot z_i}{f} \end{aligned} \qquad [1]$$

where the focal length f, the principal point coordinates c_x and c_y are derived from the intrinsic matrix K. T is the baseline distance between the two cameras determined from the extrinsic parameters[4,5].

Supporting Note 2: Aortic Valve Model

The aortic valve model is based on a comprehensive three-dimensional parametric geometry for biomechanical modeling[6]. The model represents the native aortic valve and its root, including cusps, commissures, and sinuses. This general mathematical description utilizes three independent parametric curves to construct the 3D structure of the valve. In our model, key geometric parameters are defined as follows: The commissure radius r_co is 15 mm, and the free edge radius r_fo is 3.24 mm. The cusp radius r_f is set to 0.84 mm, while the height of the free edge h_f is 4.2 mm. The height of the leaflet h_1 is 7.2 mm, and the ventricular radius r_v is 12 mm. These parameters were chosen to represent typical dimensions observed in healthy valves (Fig. S1).

(a)

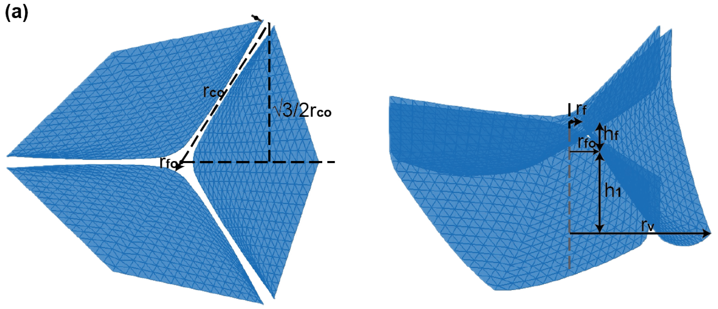

(b)

Parameter	Dimension
rco	15
rfo	3.24
rf	0.84
hf	4.2
h1	7.2
rv	12

Figure S1. Aortic valve model and the parameters. (a) Aortic valve model in the top view and front view. (b) The corresponding parameters and values are used in this paper.

Supporting Note 3: Spatial Abnormality Generator

In this work, pore shapes, the 2D periodic complex microstructures, are used to generate various abnormal shapes[7]. Starting with the basic circular pore shape, Fourier series expansion is employed to modify the contours according to the following equations:

$$
\begin{aligned}
R_\theta &= R_0\left[1 + C_1\cos(4\theta) + C_2\cos(8\theta)\right] \\
x_1 &= R_\theta \cos(\theta) \\
x_2 &= R_\theta \sin(\theta)
\end{aligned} \qquad [2]
$$

where $0 \le \theta \le 2\pi$, and three parameters R_0 (controlling the base size), C_1, and C_2(modifying the shape) have been introduced. By rotating around the z-axis, a variety of 3D shapes can be obtained (Fig. S2).

Supporting Note 4: Estimation of ground-truth in experimental examples

The comparison of tracking key points' displacements and the estimated ground-truth displacements for the two models with and without polyimide membrane. The dashed lines representing the tracking displacements align closely with the solid lines representing the numerically estimated displacements (Fig. S3).

Supporting Note 5: Additional beam bending test

To demonstrate the generality of our method, we introduce an additional new case for the beam bending test, which shares the same geometry, boundary conditions, and tracking points as the predefined benchmark model but uses a different Saint Venant-Kirchhoff constitutive model.

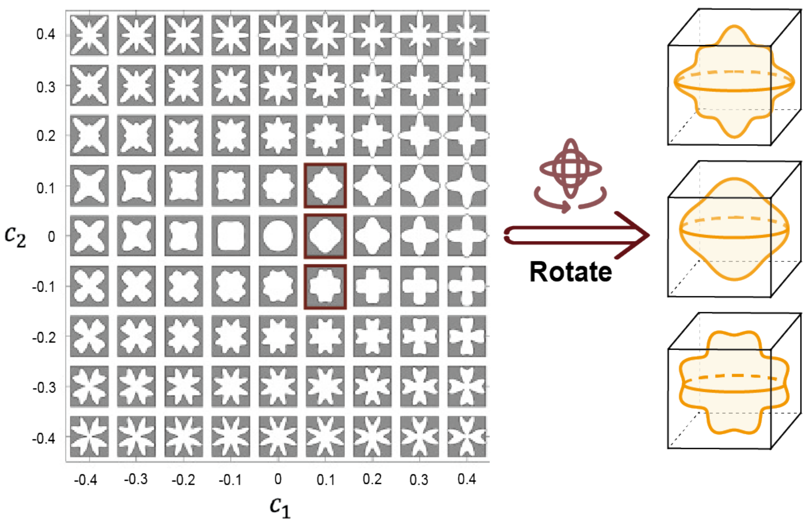

Figure S2. Spatial abnormality generator using pore shapes. Pore shapes with different combinations of parameters C_1 and C_2. And the generated 3D shapes by rotating around z-axis.

The Saint-Venant-Kirchhoff model is a hyperelastic material model that extends the geometrically linear elastic material model into the geometrically nonlinear regime. This model is widely used in solid mechanics, particularly for describing the elastic behavior of materials under small deformations. The Lagrangian Green strain $\mathbf{E}$ is given by:

$$\mathbf{E} = \frac{1}{2}\left[(\nabla\mathbf{u})^T + \nabla\mathbf{u} + (\nabla\mathbf{u})^T \cdot \nabla\mathbf{u}\right] \qquad [3]$$

where $\mathbf{u}$ is the displacement field, and ∇ is the gradient operator in the reference configuration. The Lamé constants λ and μ define the material's stiffness properties. The strain-energy density function for the Saint-Venant-Kirchhoff model is given by:

$$W(\mathbf{E}) \;= \frac{\lambda}{2}\left[\mathrm{tr}(\mathbf{E})\right]^2 + \mu\,\mathrm{tr}(\mathbf{E}^2) \qquad [4]$$

and the second Piola-Kirchhoff stress can be derived from the strain-energy function by:

$$\mathbf{S} \;= \frac{\partial W}{\partial \mathbf{E}} \qquad [5]$$

The relationship between the Lamé constant μ and Young's modulus E is as follows:

$$E = 2\mu(1+\nu) \qquad [6]$$

The displacement field calculated by the finite element simulation using the neo-Hookean model is taken as the target for this estimation. The estimated variables are the same as those in case 3 of the beam bending test: Young's modulus of the abnormal material $\tilde{E}_a$, along with the central position $\tilde{C}$ and half-width $\tilde{W}$ of the abnormality. The test converges to nearly real values of material properties within no more than 10 iterations, with a 0.31% error, which is relatively small. (Fig. S4) Therefore, we show that our method can accurately recover the correct material properties and their position using two different models, effectively illustrating the generalizability and robustness of our method.

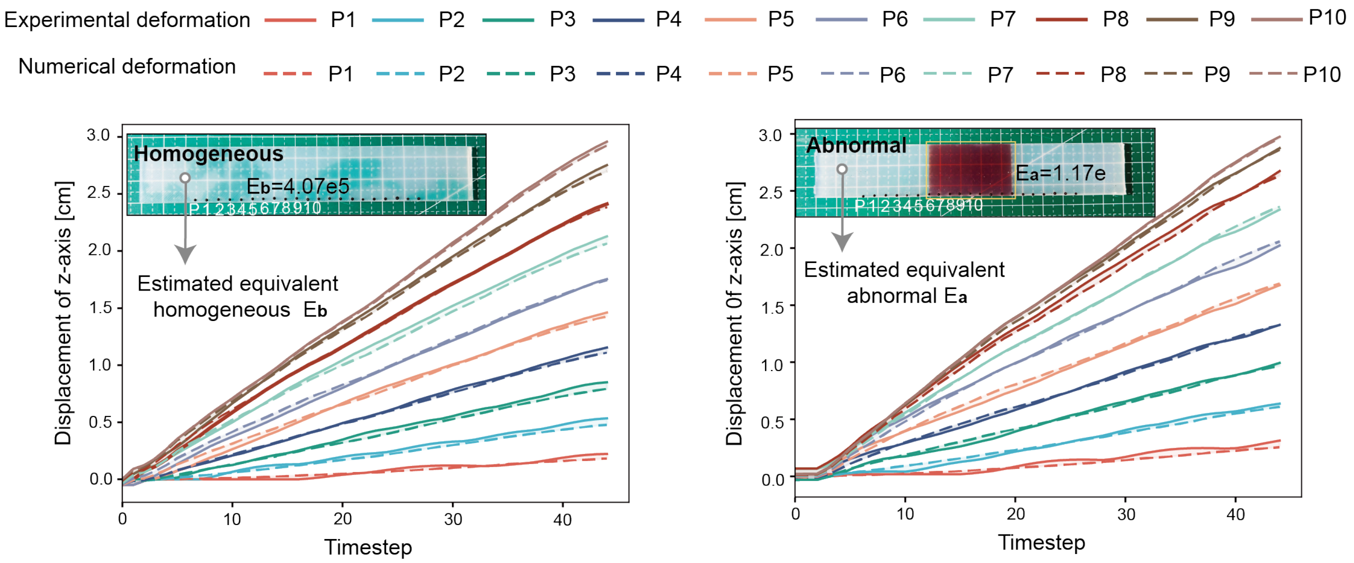

Figure S3. Results of estimated ground-truth in experimental experimental examples. The comparison between the tracking and estimated displacements for two models. The dashed lines are the tracking displacements and the solid lines are the numerical estimated displacements.

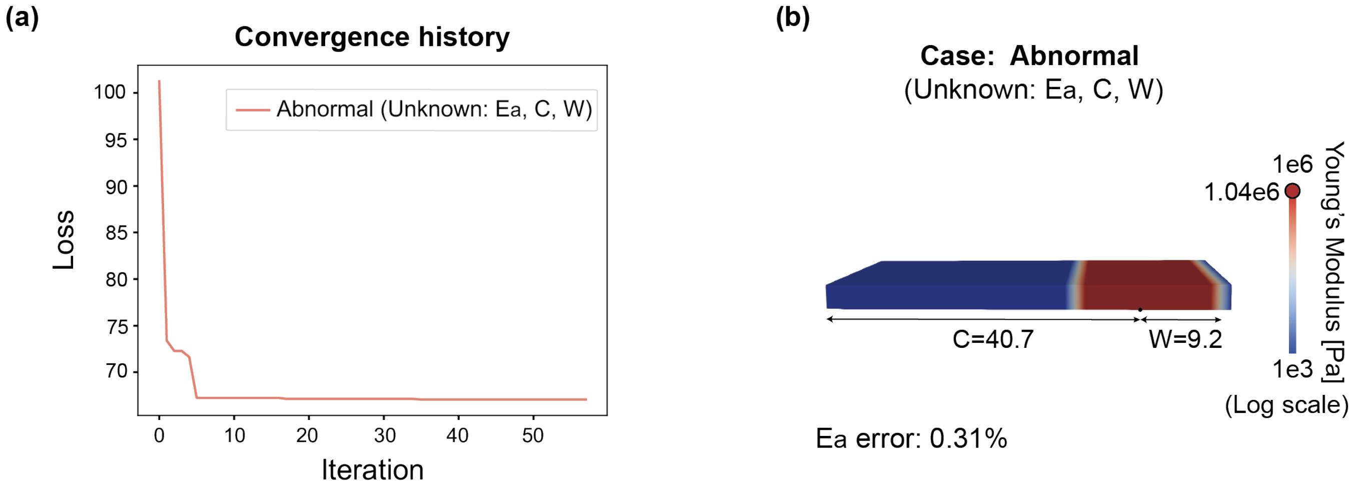

Figure S4. Results of the additional beam bending test. (a) Convergence history of the test case. (b) E_b is known, geometry and material properties of the abnormal part are to be estimated.

References

1. Bernardino, A. & Santos-Victor, J. Binocular tracking: integrating perception and control. *IEEE Transactions on Robotics Autom.* **15**, 1080–1094 (1999).

2. Islam, A., Asikuzzaman, M., Khyam, M. O., Noor-A-Rahim, M. & Pickering, M. R. Stereo vision-based 3d positioning and tracking. *IEEE Access* **8**, 138771–138787 (2020).

3. Zhang, Z. A flexible new technique for camera calibration. *IEEE Transactions on pattern analysis machine intelligence* **22**, 1330–1334 (2000).

4. Szeliski, R. *Computer vision: algorithms and applications* (Springer Nature, 2022).

5. Hartley, R. & Zisserman, A. *Multiple view geometry in computer vision* (Cambridge university press, 2003).

6. Haj-Ali, R., Marom, G., Zekry, S. B., Rosenfeld, M. & Raanani, E. A general three-dimensional parametric geometry of the native aortic valve and root for biomechanical modeling. *J. biomechanics* **45**, 2392–2397 (2012).

7. Overvelde, J. T. & Bertoldi, K. Relating pore shape to the non-linear response of periodic elastomeric structures. *J. Mech. Phys. Solids* **64**, 351–366 (2014).
